# Designing instrument to measure STEM teaching practices of Malaysian teachers

**DOI:** 10.1371/journal.pone.0268509

**Published:** 2022-05-20

**Authors:** Mageswary Karpudewan, Pavitra Krishnan, Mohd Norawi Ali, Lay Yoon Fah

**Affiliations:** 1 School of Educational Studies, Universiti Sains Malaysia, Penang, Malaysia; 2 Faculty of Psychology and Education, Universiti Malaysia Sabah, Sabah, Malaysia; University of Eastern Finland: Ita-Suomen yliopisto, FINLAND

## Abstract

The remarkable upsurge in the attention for STEM education globally has inspired many countries including Malaysia to formulate STEM education policies to reform the existing segmented teaching of the four STEM subjects towards integrated teaching. One of the Malaysian government’s initiatives include establishing a framework as a guide for teachers to practise STEM teaching. This exploratory, mixed methods study aimed to explore Malaysian science and mathematics teachers’ perceptions to practise STEM teaching and develop a questionnaire to measure factors that explain their teaching practices. The interview findings identified teachers’ knowledge of interdisciplinary and related pedagogical strategies, challenges encountered in STEM teaching practices, and teachers’ self-efficacy beliefs to perform STEM teaching as factors that explain STEM teaching practices. Following that, a 33-item questionnaire was developed based on the qualitative findings. The results of exploratory factor analysis produced four distinct factors echoing the qualitative findings with 29 items, which were then validated using confirmatory composite analysis (CCA). CCA results in retaining all four factors and removing six items with lower loading values. Thus, the final version of the questionnaire consists of 23 items. The findings of this study were expected to benefit STEM advocates and educators globally. Additionally, the developed questionnaire would allow collective measurement of the factors that explain STEM teaching practices.

## Introduction

The growing interest in science, technology, engineering, and mathematics (STEM) globally has prompted numerous countries to transform their segmented teaching of STEM subjects into integrated teaching through the formulation of STEM education policies [1,2]. The policies directed the execution of integrated STEM education to train students to be more prepared for multidisciplinary career demands and equip them with necessary skills and knowledge of STEM to deal with everyday challenges and complexities, including climate change and public health [3]. An educational reform typically involves transforming and standardising the curriculum content, pedagogical information, tests and examinations, and learning assessment [4]. Like other educational reforms, STEM educational reform involves various frameworks and approaches [3,5–7]. With the global economic challenges and demands today, the lack of STEM skills and knowledge among students who are part of the future workforce [8,9] emphasises the significant need for STEM education reform in Malaysia, where this study was performed. Like any other developing country, the national agenda in Malaysia promotes STEM education, considering the declining interest and motivation of students towards STEM learning [10]. The Ministry of Education (MoE) established a STEM education framework to guide Malaysian teachers to integrate STEM teaching in the classroom [11].

### STEM teaching practice of Malaysian teachers

**Fig 1:**
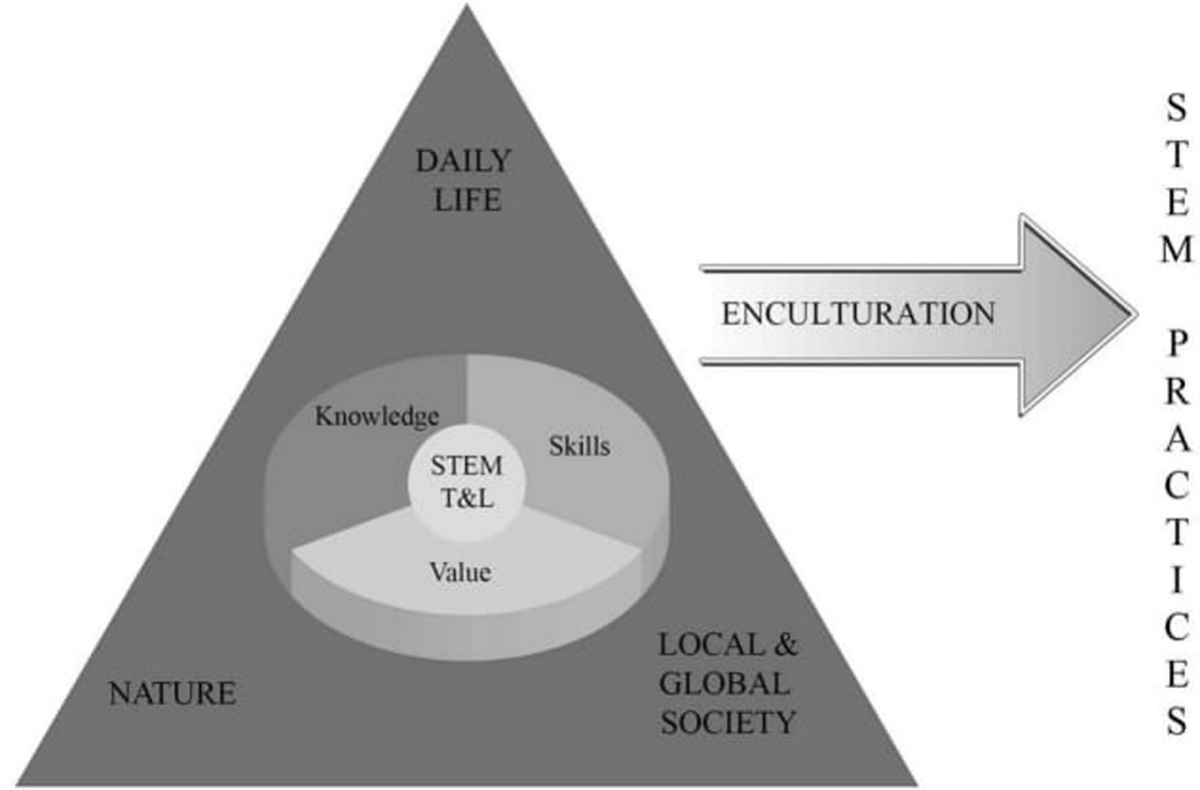
Framework for STEM teaching practices [15].

Sanders [12] defined STEM education as a purposeful combination of science, technology, engineering, and mathematics concepts and approaches. English [1] described STEM education as developing 21st-century skills and acquiring content knowledge of science, technology, engineering, and mathematics. Meanwhile, Vasquez et al. [5] suggested different levels for incorporating the four disciplines in the teaching and learning of core disciplines and skills. On a similar note, Honey et al. [13] defined STEM education as integrated teaching and learning of four disciplines concerning real-world problems. The definitions and descriptions of STEM education from the literature [5,7,13,14] guided the policymakers in MoE to propose an integrated STEM teaching framework as in Fig 1 [15].

The circle in the middle of the triangle suggests that STEM teaching and learning in Malaysia educate the students on interdisciplinary knowledge, value, and skills. The triangle edges recommend three different platforms for teachers to contextualise the lessons to bring interdisciplinary perspectives to the classes. The policymakers further suggested that project, problem, and inquiry-based learning are effective strategies to contextualise the science or mathematic lessons by incorporating knowledge, skills, and values for enculturing STEM practices. As in Fig 1, the framework has been included in the science, mathematics, and design and technology curriculum specifications across primary to secondary levels as a guide for the teachers teaching the three subjects to plan and design STEM instruction distinctively. Integrating the framework within the curriculum specifications of the three subjects signifies the MoE’s intention to urge the teachers of the three subjects to transform the learning objectives of the subjects into interdisciplinary STEM teaching and learning. The teachers of the three subjects were encouraged to practise STEM teaching to achieve the lessons’ aims. The framework that intended to transform the conventional discipline-specific into interdisciplinary compels using science and mathematics subjects as the anchor disciplines and the other disciplines as integrators resembles Moore’s Framework for STEM Integration in the Classroom [7].

Unlike other educational reforms, it is challenging to pursue STEM educational reform due to teachers’ difficulties in transforming the policies into actual practices [16–19]. Numerous studies have noted the uncertainties and indecisiveness of teachers to practise integrated teaching for STEM subjects in the classroom [16,17,20–24]. For example, Saudi Arabian teachers feel that they lack knowledge of STEM disciplines and thus are less prepared to teach in an interdisciplinary manner [21]. Besides feeling less informed about interdisciplinary pedagogical knowledge, Saudi Arabian teachers also experience low self-efficacy as well as lack of administrative and parental support and encounter various difficulties, including time constraints, lack of resources, and absence of training. A report on the challenges to STEM education in the Asia Pacific discovered that self-efficacy affects teachers’ participation in integrated STEM teaching in China, Hong Kong, and Taiwan [25]. Furthermore, Korean teachers feel that time constraints, heavy workload, and missing administrative and financial support are the deciding factors in effectively practising science, technology, engineering, arts, and mathematics (STEAM) education an approach of including arts to the STEM teaching [23]. Studies of teachers from Western countries show that integrating STEM and the associated classroom practices determines how well teachers perform [16]. The support teachers received from school administrators and parents underscores the successful implementation of STEM teaching besides interdisciplinary knowledge and self-efficacy [17]. Preparedness, self-efficacy, and attitude are the factors that inform elementary teachers’ participation in STEM teaching [22]. In addition to many context-specific factors, the above-mentioned qualitative literature established common characteristics such as teachers’ knowledge of interdisciplinary and related pedagogical strategies, challenges encountered, and teachers’ self-efficacy to conduct STEM teaching. However, studies documenting factors that inform Malaysian teachers practising STEM teaching are scarce. In the following sections, we reviewed the literature to understand how teachers’ lack of knowledge about interdisciplinary and related pedagogical strategies, encountered challenges, and their level of self-efficacy affected them in practising STEM teaching in the classroom.

## Knowledge of interdisciplinary and related pedagogical strategies

As teacher training courses only provide teachers with discipline-specific pedagogical knowledge and content knowledge [22], teachers eventually possess limited knowledge to conduct integrated STEM teaching [22,26]. Teachers have an inadequate understanding of STEM teaching practices despite agreeing that STEM teaching practices offer various opportunities for students to learn science and mathematics in a more relevant and meaningful manner [26–28]. Studies have recommended providing teachers with continuous learning and development opportunities through professional training courses [3,22] to equip them with pedagogical strategies related to STEM teaching practices [29]. The studies called to expose teachers to pedagogical approaches such as engineering design thinking, inquiry-based teaching, project, and problem-based learning to establish effective education delivery from multifaceted perspectives through integrating STEM.

### Challenges in practising STEM teaching

Effective execution of STEM education requires a strategic approach [6] that may be challenging for teachers to apply [29]. There are various challenges in practising STEM teaching due to the multifaceted perspectives of integrated STEM education such as the lack of resources and inadequate quantity and quality of STEM curriculum [16]. For instance, Peterman et al. [26] identified the lack of quality instructional materials that explicitly link science, technology, engineering, and mathematics and support for STEM learning outcomes as the utmost challenges for teachers to practise STEM teaching practices. Teachers need to spend a significant amount of time and effort to design an effective interdisciplinary STEM teaching pedagogy for their students [25]. However, teachers do not have the time to plan and prepare interdisciplinary STEM teaching materials because they are frequently overwhelmed with their existing teaching tasks [28].

### Teachers’ self-efficacy beliefs to perform STEM teaching

Self-efficacy denotes teachers’ beliefs in their preparedness to effectively execute teaching to affect students’ learning positively [30]. Teachers’ self-efficacy is highly significant in realising successful STEM education [17]. Teachers’ self-efficacy beliefs are both context- [31] and content-specific [32]. Nadelson et al. [22,32] found a positive relationship between the perceived self-efficacy of teachers and the use of inquiry in STEM education. Teachers’ self-efficacy in STEM education also affects their willingness to execute STEM teaching [28]. Based on a sample of Taiwanese teachers, teachers’ perceived self-efficacy in STEM knowledge influences their attitudes towards STEM education [25]. In another study involving a sample of Chinese teachers, teachers’ self-efficacy in STEM teaching positively influenced teaching competencies and learning outcomes [33]. Studies conducted among Saudi Arabian [20,21] and Korean [23] teachers reported low self-efficacy as a major factor in affecting STEM teaching practices. Further, a study with Hong Kong teachers depicts those teachers are not ready to teach STEM due to lower self-efficacy. The integral role of self-efficacy prompted Kelley et al. [34] to conduct a professional development course known as Teachers and Researchers Advancing Integrated Lessons in STEM (TRAILS) to improve the teachers’ self-efficacy to practise STEM teaching.

### Questionnaire to quantitatively explore STEM teaching practice

Predominately, teachers’ perceptions of STEM teaching practices were explored qualitatively [9,10,13,14,32]. The studies exhibit several limitations. El-Deghaidy et al. [20] performed the study with a small group of teachers (N = 21) from one district. Wang et al. [16] and Stohlman et al. [17] employed a case study approach and constant comparative method to analyse various data sources to reach conclusions on teachers’ perceptions of STEM teaching. Leung [29] analysed 15 students’ work to understand how inquiry-based learning, mathematical modelling, and tool-based pedagogy go hand in hand to provide an integrated STEM pedagogical framework. The above studies lack generalisation because the studies involve a limited number of specifically identified teachers, cases, or students. If the studies were conducted with larger samples using questionnaires, perhaps the findings would adequately provide recommendations to the policymakers and curriculum developers. In a different study, Park et al. [23] identified five themes reflecting the early childhood teachers’ STEM teaching practice from the responses to the open-ended online survey provided by 830 teachers. The study exhibits good generalisation because of the larger sample size [34]. However, analysing the open-ended responses of a large sample is tedious. The research would have been less demanding if the researchers had used a closed-ended questionnaire with items presented in Likert scales.

Park et al. [23] performed an online questionnaire study to explore Korean teachers’ STEAM teaching. A total of 729 teachers practising STEAM teaching in Korea responded to the questionnaire. The findings are generalisable because the study was conducted using a large sample size. However, the questionnaire used exhibits limitations. The questionnaire consisted of two sections. The first section, with three self-reported questions of “How many times they taught STEAM lessons, type of curriculum, and subject used to integrate STEAM”, measured STEAM teaching practice. The second section on teachers’ perceptions of STEAM education comprised of 3 subscales: overall perception, potential impact, and challenges faced. The overall perception evaluated teachers’ views on the importance of STEAM education. The subscale “potential impact” measured teachers’ opinions on the positive effects of STEAM education on thinking skills. The third subscale on the challenges faced included specific items measuring difficulties such as lack of support, time, increased workload, and other problems encountered by the teachers. With the exception of challenges, the other two subscales focused on measuring the outcomes of STEAM education for students. These two subscales have a limited association with teachers’ views on practising STEAM. The items and subscales do not explicitly measure the factors that explain STEAM teaching.

A few other studies used questionnaires to measure the teachers’ self-efficacy [16,24,25] as well as knowledge and perception [22,34]. These studies did not explore the factors that explain STEM teaching practices. Instead, the studies focused on measuring teachers’ self-efficacy, knowledge of STEM, and their perceptions from participating in a STEM workshop. Lee et al. [25] and Nadelson et al. [22] used a questionnaire developed initially by Bandura [35] to measure self-efficacy. Yoon et al. [31] developed a set of items to measure teachers’ efficacy in teaching engineering. For measuring knowledge, Nadelson et al. [32] created items measuring teachers’ knowledge of using the inquiry approach as a platform to perform integrated STEM teaching. The review shows the absence of a questionnaire that collectively measures the factors that inform teachers’ STEM teaching practice. Such a questionnaire is imperative for Malaysia as the questionnaire would allow gathering data from a larger sample of teachers to advise the STEM education curriculum developers and policymakers on the way forward.

## Aims of the study

The current study was conducted to bridge the gap in information about factors that influence Malaysian teachers practising STEM teaching and absence of questionnaire to evaluate the factors. The following objectives guided the current study:

1. To identify the key factors that explain STEM teaching practices of Malaysian science and mathematics teachers.
2. To develop a specific questionnaire to measure factors that explain their STEM teaching practices.

## Methods

### Research design

**Fig 2:**
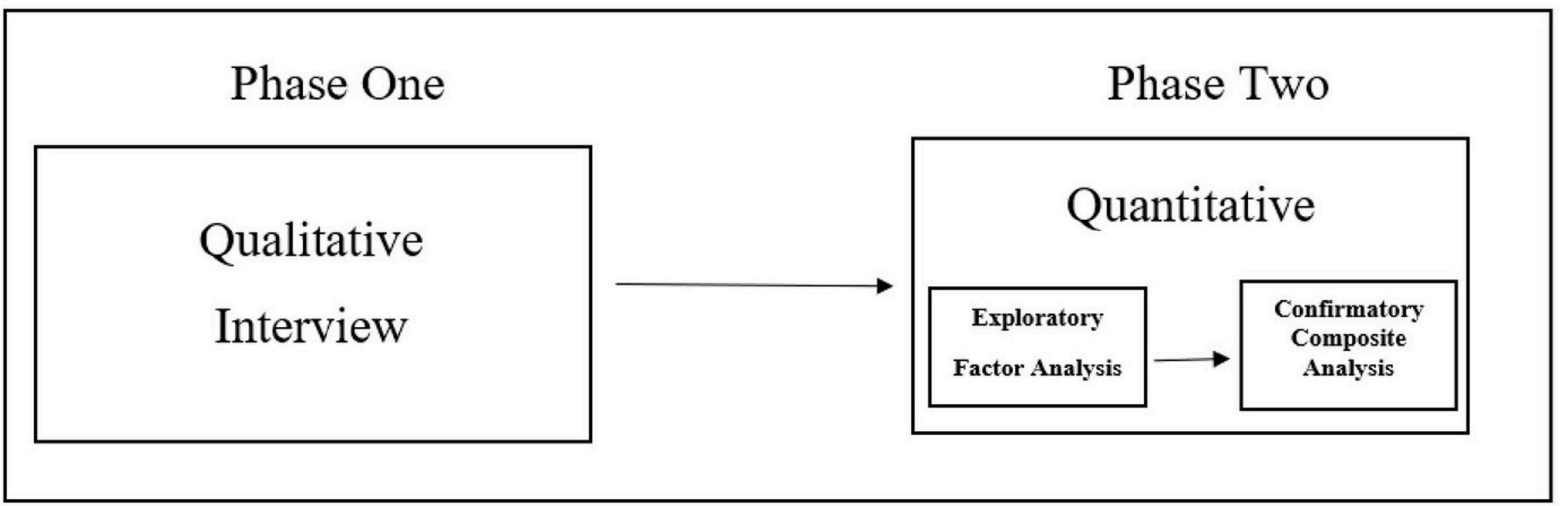
Exploratory mixed methods research design employed in this study.

Creswell [36] recommends using exploratory mixed methods research design to explore any problem and take the exploration findings to develop an instrument or treatment. The exploratory mixed methods research design entails collecting and analysing qualitative data in the first phase. In the second phase, which is the quantitative phase, the qualitative findings inform the development of an instrument. Fig 2 illustrates the adaptation of exploratory research design in this research. As shown in Fig 2, this study was conducted in two phases (qualitative and quantitative phases).

The design is the most appropriate design to accomplish the research objectives of the current study. The interpretivist perspective and constructivism theory guided in deciding the methodology for the qualitative phase (phase one) of the study. The first research objective of the study describes the knowledge of STEM teaching practice constructed by the individual teachers from their experiences. The purposively identified Malaysian science and mathematics teachers were individually interviewed and the interview narratives were analysed to identify the knowledge. The second research objective proposed to use the qualitative findings from the first research objective to develop a questionnaire that can be used to measure the factors.

In the quantitative phase (the second phase), first, we constructed questionnaire items based on the interview responses. We constructed the items according to the themes from the qualitative interview responses. Then we conducted exploratory factor analysis (EFA) to discover the factor structures and confirmatory composite analysis (CCA) using partial least squares structural equation modelling (PLS-SEM) [37] to confirm the factor structures. Although confirmatory factor analysis (CFA) is typically used to reaffirm the underlying factor structure and the corresponding items [30,38], CCA is proposed as an alternative to CFA to measure confirmatory factors [39].

### Sampling and data collection procedure

For the qualitative phase of the study, we employed a random purposive sampling strategy to recruit the 40 (19 males and 21 females) science and mathematics teachers who participated in the interview. We used a purposive sampling strategy because the study focused exclusively on science and mathematics teachers. The STEM teaching framework, as in Fig 1, infers transforming the discipline-specific learning objectives of science, mathematics, or design and technology into interdisciplinary perspectives. The integration implies that science, mathematics, and design and technology subjects are the anchor disciplines and other disciplines are integrators. In this way, the teachers of the three subjects perform STEM teaching in accordance with the objectives of the individual subjects. In Malaysia, science and mathematics teachers were given heightened importance for practising STEM. The 40 teachers were randomly identified from the name list of science and mathematics teachers provided by the state education department of the 13 states in Malaysia. The 40 teachers are teaching science and mathematics at primary and secondary schools across the 13 states in Malaysia. Of the 40 teachers, 23 teach science and mathematics to primary-level students. The rest (17) were employed in secondary schools teaching general science, physics, chemistry, and biology. The samples exhibit almost identical gender distribution. The teaching experience ranging from 4 to 20 years suggests that the sample consists of junior and senior teachers.

Different science and mathematics teachers have participated in the factor analysis (EFA and CCA). The participating teachers were recruited using a purposive random sampling strategy. Purposive reflects the analysis solely involved science and mathematics teachers. These teachers were randomly identified from the list of teachers provided by the state education department from all the 13 states in Malaysia. The teachers were invited through email to explain the purpose of the study. Upon accepting the invitation, the questionnaire was emailed to 372 teachers. Only 321 teachers returned the questionnaires. We had to discard 21 incomplete questionnaires. The rest, 300 (180 female and 120 male) responses, were used to perform the EFA. The sample size (n = 300) is the recommended size for EFA (Hair et al., 2017). A total of 55 respondents were between 45 and 60 years of age, and another 149 respondents were between 30 and 44 years old. The remaining respondents aged between 25 and 39.

The CCA was conducted four months after performing EFA. The science and mathematics teachers who participated in CCA are different from those who participated in EFA. Like EFA, we identified the participants for CCA (n = 397) using a purposive sampling strategy. Based on the results of the G*Power calculator, a minimum sample size of 103 was suggested. Therefore, the acquired sample size (n = 397) was adequate. About 64.5% of the total respondents were female science and mathematics teachers, while 35.5% were male science and mathematics teachers. These respondents were mainly between 31 and 41 years old (38.8%), followed by those between 36 and 40 years old (26.2%), between 41 and 45 years old (9.8%), between 51 and 56 years old (8.9%), between 46 and 50 years old (8.1%), and between 26 and 30 years old (6.5%). The remaining respondents were above 55 years old (1.7%). Besides that, the majority of the respondents were from secondary schools (59.2%). The remaining respondents were from primary schools (40.8%). Additionally, 64.7% of them reported teaching science and mathematics in schools located in the urban areas. The remaining respondents were science and mathematics teachers in rural areas (35.3%).

### Measures

To accomplish the study’s research objectives, we employed two different measures: qualitative semi-structured interviews and a questionnaire. Like the earlier studies [9,10,13,14], we adapted the semi-structured interview method to identify the factors. The earlier studies guided the formulation of the interview questions. Two local STEM experts and three experienced STEM teachers validated the questions. Based on the experts’ feedback, the interview questions were revised and structured as presented in S1 Appendix.

The questionnaire used in this study is developed by the researchers from analysing the interview responses. The data analysis section provides the details on how we constructed the items as well as explored and confirmed the factor structures.

### Data analysis procedure

#### Interview data analysis

All interview responses were recorded, transcribed, and analysed according to the thematic analysis framework proposed by Braun and Clarke [40]. First, the recorded data were transcribed. The transcripts were read several times in the second step for data familiarisation. A total of 18 codes were extracted and grouped into three main categories: (1) teachers’ knowledge of interdisciplinary and related pedagogical strategies, (2) challenges that limit STEM teaching practice, and (3) self-efficacy to perform STEM teaching. In the next step, the initial codes and established categories were re-examined to ensure the accuracy of data categorisation for all extracted codes. These three categories were found consistent with the documented findings from teachers’ perceptions of factors that explain their STEM teaching practices in the classroom [16,17,20–23]. As shown in S2 Appendix, a specific theme was derived from merging the codes and categories in the final step. Previously appointed experts also participated in the data analysis. The inter-rater reliability was between 0.67 and 0.89, suggesting a substantial agreement among the experts [39].

#### Constructions of questionnaire items

Based on the interview findings from the qualitative phase and past studies [16,17,20,21], a questionnaire measuring factors that explain STEM teaching practices was developed in the quantitative phase. The derived theme, categories, and codes in the qualitative phase and the past studies [16,17,20,21] led to the formation of four constructs: (1) STEM teaching practices (STP), (2) teachers’ knowledge of interdisciplinary and related pedagogical strategies (KN), (3) challenges that limit STEM teaching practices (CH), and (4) teachers’ self-efficacy beliefs to perform STEM teaching (PSE). A total of 33 items using five-point Likert scales were constructed to measure STEM teaching practices of Malaysian science and mathematics teachers: (1) STP construct consisted of 10 items (STP1 to STP10), (2) KN construct consisted of nine items (KN1 to KN9), (3) CH construct consisted of 10 items (CH1 to CH10), and (4) PSE construct consisted of four items (PSE1 to PSE4). Teachers are required to indicate to what extent they agree to the statements in scales of 1 (strongly disagree) to 5 (strongly agree).

These items were constructed based on the analysis of interview responses for each interview question. For instance, STP2 (“*I ensure students work collaboratively in groups during STEM teaching*”) was derived from the obtained interview response (“*STEM teaching engages students to work in groups*”) for the interview question of “*How do you practise STEM teaching in the classroom*?”. The response to the importance of engineering (“*Engineering is an important aspect in STEM education*”) for the question of “*What do you know about STEM education*?” led to the formulation of STP9 (“*Engineering is one of the important components of STEM education that teach students to apply the knowledge of science and mathematics*”). Meanwhile, the mention of limited resources for the interview question on the difficulties in STEM teaching practices led to the construction of CH3 (“*inadequate quantity of instructional materials for STEM teaching*”). There were also mentions of having low confidence to perform integrated STEM teaching among the interview participants, which led to the formation of PSE4 (“*I am confident to deliver integrated STEM education*”). The rest of the questionnaire items were constructed similarly.

#### Factor analysis

The 300 responses to the questionnaire developed were subjected to EFA in three steps. First, with the guidance of literature, we decided to extract five factors from the data set. Next, we extracted the factors using principal component analysis. Finally, we used the varimax rotation method to rotate the factors to demonstrate independent relationships among the extracted factors. In the analysis, items with loading values of less than 0.50 and cross-loaded on two or more factors at 0.50 or higher were excluded. Second, the eigenvalue of 1.0 was used as the cut-off value. Items with eigenvalues of less than 1.0 were extracted. The CCA focused on validating the obtained EFA results through CCA using PLS-SEM [33]. A five-step CCA is proposed for such purposes: estimation of loadings and significance, indicator reliability of the items, composite reliability (CR) of the constructs, average variance extracted (AVE), as well as discriminant validity, nomological validity, and predictive validity.

### Ethical approval

This study was performed in line with the principles of the Declaration of Helsinki. Approval was granted by the Ethics Committee of Universiti Sains Malaysia (USM/JEPeM/19090532). The committee reviewed and approved the research design and the data collection approaches of the current study. The participants agreed to participate voluntarily and could withdraw from the study freely at any time. The data were confidential and participation was anonymous without any potential risk. All study participants provided informed consent to participate in the study and publication of the data.

## Results

### Interview findings

Based on the obtained interview findings, three factors that explain STEM teaching practices of science and mathematics teachers were identified. These factors are discussed in the following subsections.

#### Teachers’ knowledge

From the viewpoints of the participating science and mathematics teachers in this study, most of them viewed STEM education as education that emphasises the teaching and learning of all four subjects of science, technology, engineering, and mathematics. Some of their responses are as follows: “*Students learn both science and math together*”, “*It is about teaching engineering during science lessons*”, and “*Technology forms the main component of teaching for students to keep abreast with the advancement in technology*”. This implies that teachers know that STEM education involves integrated teaching and learning of these subjects.

Despite that, the participants did not highlight the interdisciplinary and related pedagogical strategies for students to apply knowledge from these four disciplines to solve real-world problems. Instead, the participants noted the following: “*Students cannot simply follow recipe-like procedures to conduct hands-on activities like what we are doing now*. *Rather they need to engage in thinking*”, “*Teaching should include higher-order thinking skills*”, and “*We don’t emphasize applying the concepts that require students to think to solve a problem*”. Based on these responses, teachers are aware of the focus of integrated STEM teaching in equipping students with the necessary skills and knowledge.

A few participants expressed their agreement on the role of STEM education in preparing the students from the early stage of schooling for their future career: “*equipping knowledge for joining the industry later*”, “*Students were taught on solving a problem so that they can apply the skills when they join the industry*”, and “*Knowing science concepts alone is not sufficient for students to secure a good job*. *They need to have engineering skills and current technological knowledge as well*. *STEM education helps them*”. These responses are consistent with the notion that STEM education trains students to meet multidisciplinary career demands as part of the future workforce.

Other notable responses from the participants are the claim that inquiry-based teaching strategy is an appropriate strategy to deliver STEM teaching in the classroom. The participants also described the significance of combining problem-based learning and inquiry-based teaching for students. Besides that, a few participants expressed the significance of introducing projects as part of the integrated STEM teaching in the classroom. These responses reflect the multifaceted perspectives of science and mathematics teachers on the strategies that influence them to practise STEM teaching in the classroom.

#### Challenges encountered

Based on the participants’ responses, the lack of time was identified as the key aspect that affects their intention to deliver integrated STEM teaching. The participants highlighted the lack of time to prepare STEM teaching materials and the short duration to deliver integrated STEM teaching from the existing schedule of teaching science and mathematics separately. Statements such as “*Integrated STEM teaching requires comprehensive planning*. *This requires referring to multiple resources to plan the teaching*” and “*quite impossible to bring the interdisciplinary perspectives into science and mathematics lesson within one-hour teaching allocated in the timetable*” suggest the restricted time allocated to the teachers to deliver integrated STEM teaching in the classroom.

Another aspect contributing to the challenges in practising STEM teaching from the participants’ viewpoints is the lack of available resources, especially on the relevance of integrated STEM teaching materials within the local context. One of the participants mentioned the following: “*The definition of STEM education is ambiguous*. *Some resources are there explaining the integration*. *However*, *the examples are less suitable for local context*”. Another participant voiced a similar point: “*Project or problem-based teaching and inquiry teaching is not foreign to us*. *However*, *few references guide us to employ those strategies to deliver interdisciplinary teaching*”. The lack of resources for teaching in terms of quantity and quality does affect the STEM teaching performance of teachers.

The difficulties of science and mathematics teachers in applying problem-, project-, and inquiry-based teaching to deliver interdisciplinary STEM teaching appear to be linked to the science or mathematics teaching training they received. The science and mathematics teachers in this study highlighted that they are “*aware of using problem-*, *project-*, *and inquiry-based science education*” but proceeded to note how they are “*less informed on how inquiry-based science education could be used in STEM education*”. They further elaborated the point as follows: “*Some of us have attended professional development courses*. *However*, *we are less competent in this aspect*. *The courses do not provide substantive information for us to transform the teaching*”. In other words, without the relevant professional training courses for these teachers, they are likely to have problems of delivering STEM teaching effectively.

Besides that, the majority of the interview participants expressed agreement about the government’s overemphasis on STEM teaching in schools. The related policy reflects the stance of the government on STEM education. However, the interview participants noted the lack of exposure to STEM teaching among the teachers: “*Information about STEM education is provided in Science Standard Curriculum and Evaluation Document*. *Other than that*, *we do not have any exposure to STEM teaching*”. The limited exposure to integrated STEM teaching among science and mathematics teachers does have substantial effects on the effectiveness of integrated STEM teaching in the classroom.

Furthermore, Malaysia’s classroom and laboratory settings are not prepared to accommodate the demands of integrated STEM teaching. The participants noted the inadequacy of laboratories, apparatuses, and equipment. Such conditions do not support teachers in delivering STEM teaching. Apart from that, the large class size discourages the teachers from using STEM teaching practices or other new approaches that promote the ability to work in a team and effective transfer of knowledge and skills.

#### Teachers’ self-efficacy beliefs

The confidence to deliver the teaching and influence learning and the willingness to learn more about STEM education reflect the teachers’ self-efficacy. The following statements suggest the lack of ease in delivering STEM teaching among the participants: “*I am not trained to teach multiple subjects*. *I am not confident enough to perform such teaching*”, “*I am not sure whether my teaching will improve students learning*”, “*I think I would not be able to provide or set a meaningful learning context for the students to understand the holistic idea embraced within STEM education*”. However, certain participants noted otherwise: “*I am responsible for the student’s learning and achievement*. *I am willing to learn to conduct STEM teaching*”, “*I will continue learning about STEM teaching so that my students will be well equipped to join the workforce in the future*”. The sense of accountability to the students’ learning outcomes and the willingness to continue improving their teaching reflect their perceived self-efficacy. The above-mentioned claims imply that teachers’ perceived self-efficacy, which can be explained in terms of confidence level, continuous learning, and sense of accountability to the students’ achievements, influences the teachers’ STEM teaching performance.

### Results of exploratory factor analysis

The results of the Kaiser-Meyer-Olkin measure of sampling adequacy (0.870 > 0.500) in this study demonstrated the suitability of the data for factor analysis. The significant results of Bartlett’s test of sphericity (χ^2^(528) = 9292.931, *p* < .05) demonstrated adequate correlations between the items. The tabulated results presented in S3 Appendix revealed four factors with a total of 29 items after the removal of four items.

A total of 10 items were clustered under Factor 1. These clusters were closely linked to the challenges science and mathematics teachers encountered in STEM teaching, suggesting the label of *perceived difficulties in STEM teaching practices* [20,21,23,28]. These items explained 38.34% of the total variance, the highest variance among all factors. Meanwhile, the extracted Factor 2 consisted of seven items that reflect the factor for knowledge. Studies have also reported a similar factor referred to as “knowledge of interdisciplinary and integration” [16,17,20,21]. Considering that, Factor 2 in this study was labelled as *knowledge of interdisciplinary and related pedagogical strategies*. This factor explained 12.42% of the total variance. On the other hand, items under Factor 3 that represented perceived self-efficacy were discussed in the literature [3,16,22,25]. Factor 3 with the label of *teachers’ self-efficacy beliefs to perform STEM teaching* explained 7.62% of the total variance. The final factor consisted of eight items that describe methods and strategies used to execute STEM teaching. Thus, the final factor with the label of *STEM teaching practices* explained 5.50% of the total variance.

### Results of confirmatory composite analysis

The survey responses from a total of 397 respondents were subjected to CCA using PLS-SEM [41]. The analysis served to verify the emerging factors and items (from Study 3). The results of outer loading, average variance extracted (AVE), and composite reliability (CR) are in Table 1. The outer loadings of items determine the indicator reliability. Items with outer loading of below 0.708 were identified for removal [42]. The removed items were from Factor 1 (four items) and Factor 4 (two items), resulting in a total of 23 items for the final version of the questionnaire. The composite reliability (CR) values exceeded the threshold value of 0.7 [43], suggesting adequate internal consistency of all items. Besides that, the values of average variance extracted (AVE) exceeded the threshold value of 0.5 [44] for all four constructs. In other words, adequate convergent validity was confirmed.

**Table 1:** Outer loading, mean, and standard deviation of items.

Constructs	Item	Loading	AVE	CR
Knowledge	KN2	0.873	0.784	0.962
	KN3	0.867		
	KN4	0.892		
	KN5	0.940		
	KN6	0.889		
	KN7	0.901		
	KN8	0.834		
Perceived difficulties	PD1	0.878	0.770	0.943
	PD2	0.919		
	PD3	0.923		
	PD4	0.929		
	PD5	0.856		
	PD7	0.721		
Perceived self-efficacy	PE1	0.841	0.780	0.934
	PE2	0.905		
	PE3	0.914		
	PE4	0.896		
STEM teaching practice	STP2	0.881	0.758	0.904
	STP4	0.836		
	STP5	0.971		
	STP7	0.894		
	STP8	0.823		
	STP10	0.873		

Table 2 presents the obtained results of heterotrait-monotrait ratio of correlations, construct reliability, and validity of the emerged factors (constructs). The recorded heterotrait-monotrait ratio of correlations between the constructs did not exceed the threshold value of 0.85 [43], suggesting adequate discriminant validity among the constructs.

**Table 2:** Heterotrait-monotrait ratio of correlations, construct reliability, and validity of factors.

	Knowledge	Perceived difficulties	Perceived efficacy
Knowledge			
Perceived difficulties	0.561		
Perceived efficacy	0.628	0.362	
STEM teaching practices	0.785	0.518	0.728

For the testing of nomological validity, the outcome variable in this study, namely STEM teaching practices, was selected based on the strong evidence of its linkage to the aspects of knowledge, difficulties, and efficacy found in the literature [16,17,20–23,25]. The interview findings align with the findings of prior studies that similarly identified knowledge, difficulties, and self-efficacy as factors that explain STEM teaching practices.

Both literature and interview findings complement the quantitative results of Study 4, in which teachers’ knowledge of interdisciplinary and related pedagogical strategies and STEM teaching practices were positively correlated (*β* = 0.705; *p* < 0.01). Besides that, literature and interview findings revealed that perceived difficulties in STEM teaching practices affect their performance, which were reaffirmed in Study 4. The quantitative results of Study 4 revealed a negative correlation between both constructs (*β* = -0.446; *p* < 0.01). On the other hand, past studies reported a positive correlation between teachers’ self-efficacy beliefs to perform STEM teaching and STEM teaching practices [16,25]. The current study also demonstrated a similar correlation between these constructs (*β* = 0.643; *p* < 0.01).

The R^2^ and Q^2^ values determine the predictive validity of the constructs [44]. In this study, all constructs explained 68.7% of the variance in STEM teaching practices (R^2^ = 0.687). The values of Q^2^ were above zero for all relationships: (1) the relationship between teachers’ knowledge of interdisciplinary and related pedagogical strategies and STEM teaching practices (Q^2^ = 0.84), (2) the relationship between perceived difficulties in STEM teaching practices and STEM teaching practices (Q^2^ = 0.163), and (3) the relationship between teachers’ self-efficacy beliefs to perform STEM teaching and STEM teaching practices (Q^2^ = 0.312). These results supported the predictive validity of all constructs. Overall, the results demonstrated nomological validity and predictive validity.

## Discussion

Based on a sample of 40 science and mathematics teachers in this study, the interview findings revealed several factors that explain STEM teaching practices of Malaysian teachers: (1) teachers’ knowledge of interdisciplinary and related pedagogical strategies, (2) challenges that limit STEM teaching practice, and (3) teachers’ self-efficacy beliefs to perform STEM teaching. These findings corroborated the findings of a recent study by Gardner and Tillotson [37], which concluded that the lack of knowledge about the interdisciplinary nature of STEM teaching among teachers affects their decisions to practise STEM teaching in the classroom. The findings are also coherent with findings of other studies conducted with teachers from various countries, which indicate teachers have limited knowledge to execute engineering education and teachers are less informed to adapt strategies such as problem-, project-, and inquiry-based teaching to practise STEM teaching [16,17,20,21].

Besides that, a few studies identified the lack of training for teachers to deliver integrated STEM teaching [3,22]. Some other studies stated that teachers are required to allocate a substantial amount of time to prepare and execute STEM teaching [23,28]. The lack of contextual resources has further challenged teachers’ attempts to deliver STEM teaching effectively [16,45]. Similar points emerged from the interview responses of the teachers. This study denoted these points as perceived difficulties in STEM teaching practices. Additionally, teachers’ self-efficacy is deemed significant given its influence on their confidence level to deliver STEM teaching in the classroom and subsequently the students’ learning. Therefore, several studies have identified self-efficacy as the most important determinant among teachers in many countries such as South Korea [23], Saudi Arabia [21], Taiwan [25], China [46], Hong Kong [47], and the United States [48]. This study similarly demonstrated self-efficacy as a significant factor that explains STEM teaching practices.

The STEM teaching practice questionnaire developed through this study addresses the gap in a measure that allows documenting the practice comprehensively. The new questionnaire exhibits several advantages over the existing questionnaire items. First, the newly developed questionnaire items were measured using a five-point Likert scale compared to self-report items used by Park et al. [23]. Second, in the questionnaire by Park et al. [23], except for the subscale on challenges, the other subscales, overall perception, and impact do not indicate the practices. Besides Park et al. [23], several other studies in the past quantitatively investigated teachers’ perceptions. Nadelson et al. [32] conducted a free-response survey using six items to measure knowledge of integrated STEM and 25 items from a science teaching efficacy belief instrument to measure self-efficacy beliefs among the teachers who participated in the I-STEM summer project. A 32-item Teaching Confidence Scale by Woolfolk Hoy and Spero [49] and a modified Science Teacher Efficacy Belief Instrument (STEBI) were used in another study [22] to examine the teachers’ confidence level and self-efficacy respectively. Kelley [34] similarly applied the modified STEBI version. Focusing on the need for content- and context-specific self-efficacy, Lee et al. [25] developed an instrument to measure perceived self-efficacy in relation to STEM knowledge among Taiwanese teachers. Yoon et al. [31] quantitatively measured teachers’ self-efficacy to teach engineering. Unlike these previous studies, this study developed a questionnaire that incorporated items which collectively measure multiple factors that explain STEM teaching practices: (1) knowledge of interdisciplinary and related pedagogical strategies, (2) perceived difficulties in STEM teaching practices, (3) self-efficacy beliefs to perform STEM teaching, and (4) STEM teaching practices.

The EFA results revealed four factors and demonstrated the need to remove four items. The remaining items and factors closely corresponded to the emerging factors from the literature and the interview findings in this study on STEM teaching practices. Based on the literature review, these four factors were identified as teachers’ knowledge of interdisciplinary and related pedagogical strategies, perceived difficulties in STEM teaching practices, teachers’ self-efficacy beliefs to perform STEM teaching, and STEM teaching practices. All items of perceived difficulties in STEM teaching practices and teachers’ self-efficacy beliefs to perform STEM teaching were retained in this study. These items fit the definition of teachers’ self-efficacy [16,23,25,34] and identified challenges [23,28] to deliver STEM teaching practices. However, KN1 on engineering as a key component of STEM education and KN9 on STEM education as an independent curriculum were removed. The removal of both items suggests the teachers’ beliefs that engineering should be incorporated with science and mathematics in STEM education. This reflects the definition of integrated STEM education [14]. Under the STEM teaching practices, both STP6 and STP9 on teaching using a textbook and laboratory settings in STEM education were removed. The removal of both items reflects the teachers’ knowledge of not limiting interdisciplinary STEM education to textbook and laboratory settings.

Following that, this study conducted CCA to confirm the remaining items and factors derived from the EFA results [49]. All items with a loading value of more than 0.704 were retained. All items of teachers’ perceived self-efficacy beliefs and knowledge of interdisciplinary and related pedagogical strategies were retained. For the construct of perceived difficulties, a total of four items were dropped. These four items measured teachers’ views on the exposure to implementing STEM teaching, time to perform STEM teaching, large class size, and facilities in the laboratory. The construct of STEM teaching practices consisted of six items after two items were dropped. These two items were “STEM education forms an integral part of my everyday teaching” and “I regularly observe other teachers performing STEM teaching”. This study successfully developed a 23-item questionnaire to measure STEM teaching practices and knowledge, perceived difficulties, and self-efficacy beliefs in relation to integrated STEM education, which was discussed in various past studies [1,13,23,34].

## Implications and limitations of study

This study qualitatively identified teachers’ knowledge of interdisciplinary and other pedagogical strategies, perceived difficulties in STEM teaching practices, and teachers’ self-efficacy beliefs to perform STEM teaching as factors that explain STEM teaching practices of science and mathematics teachers. Based on the interview findings, this study developed a 33-item questionnaire. The obtained quantitative data were then subjected to EFA. The results revealed the need to remove a total of five items. Four factors were extracted and 28 items were retained. Following that, CCA was performed. All four factors were retained, but another seven items were subjected to removal due to their low loading values. With that, a 21-item questionnaire was finally developed in this study.

The study exhibits several implications. First, the findings of the study are informative for the MoE to design integrated STEM education curriculum for students and developing training materials for the teachers. The challenges as such as time constraints and limited availability of resources convey the message that the curriculum developers should provide teaching materials that enable teachers to execute STEM teaching within the time specified in the school timetable. For training materials, the findings inform the curriculum developers to consider including information about interdisciplinary teaching and the associated pedagogical strategies. Teachers should be trained to plan and execute the lessons within the time frame and the training should be aimed at improving self-efficacy of the teachers. Second, the development of a questionnaire in this mixed methods study benefits STEM advocates and educators to obtain valuable insights on teachers’ knowledge of interdisciplinary and related pedagogical strategies, perceived difficulties, and perceived self-efficacy beliefs to perform STEM teaching. The developed questionnaire in this study also addressed the gap in the availability of an instrument which can collectively measure key factors that explain STEM teaching practices. Third, this study was among the first in the educational domain to incorporate CCA. To date, only such studies have been found in the business domain [50].

The study encountered several limitations. First, studies have identified institutional support as one of the key factors in influencing STEM teaching practices [16,21] but this study revealed otherwise. This study had insufficient evidence to support this particular finding based on the obtained interview findings. Therefore, it is recommended for future research to utilise probing interview questions in gaining more in-depth responses to the support teachers receive to conduct STEM teaching. Besides that, this mixed methods study explored and established factors that explain STEM teaching practices, but there was no evidence of the influence of knowledge, difficulties, and self-efficacy on STEM teaching practices. Hence, it is recommended for future research to measure the relationships of these factors with STEM teaching practices among science and mathematics teachers.

## Supporting information

S1 Appendix (DOCX)

S2 Appendix (DOCX)

S3 Appendix (DOCX)

S1 Dataset (XLSX)

S2 Dataset (XLSX)

S3 Dataset (DOCX)
