# Supplementary material for: Designing instrument to measure STEM teaching practices of Malaysian teachers
Source: PLoS One. 2022 May 20;17(5):e0268509. doi: 10.1371/journal.pone.0268509 (PMC9122257; doi:10.1371/journal.pone.0268509)
Supplement: S1 Appendix — (DOCX) [file pone.0268509.s001.docx]

**Appendix 1**

**Interview Questions**

1. Tell me what you know about STEM education?
2. What is integrated STEM education?
3. Is integrated STEM education different from the existing science and mathematics subjects?
4. Do you practice STEM teaching? How often?
5. What are the examples of integrated STEM activities?
6. Do you encounter difficulties in obtaining STEM teaching materials?
7. Does your lab provide sufficient infrastructure to carry out STEM teaching?
8. Do you have enough training, or have you attended any training on STEM education?
9. Do you believe that you have the confidence to deliver STEM education?
