# Supplementary material for: Designing instrument to measure STEM teaching practices of Malaysian teachers
Source: PLoS One. 2022 May 20;17(5):e0268509. doi: 10.1371/journal.pone.0268509 (PMC9122257; doi:10.1371/journal.pone.0268509)
Supplement: S2 Appendix — (DOCX) [file pone.0268509.s002.docx]

**Appendix 2**

**Theme, categories, and codes derived from interview responses**

| Codes | Categories | Theme |
| --- | --- | --- |
| STEM domains and definition  STEM careers  STEM activities and approaches  STEM is about technology.  Engineering is an important aspect of STEM.  STEM aims to develop skills among students.  STEM helps students to solve daily life problems.  Difference between STEM and the current science and mathematics lessons | Teachers' knowledge of interdisciplinary and related pedagogical strategies | Factors that explain STEM teaching practices |
| Lack of infrastructure  Additional workload for teachers  Large class size  Lack of exposure  Lack of training  Lack of resources | Challenges that limit STEM teaching practices |  |
| Teachers’ confidence  Responsible for enhancing students’ learning  Willingness to improve themselves | Teachers' self-efficacy beliefs to perform STEM teaching. |  |
