# Supplementary material for: Designing instrument to measure STEM teaching practices of Malaysian teachers
Source: PLoS One. 2022 May 20;17(5):e0268509. doi: 10.1371/journal.pone.0268509 (PMC9122257; doi:10.1371/journal.pone.0268509)
Supplement: S3 Appendix — (DOCX) [file pone.0268509.s003.docx]

**Appendix 3**

**Factor structure and corresponding items derived from EFA**

| Items | Factor | | | |
| --- | --- | --- | --- | --- |
|  | 1 | 2 | 3 | 4 |
| Factor 1: *Perceived difficulties in STEM teaching practices*  PD4: Inadequate availability of quality STEM resources  PD7: Lack of training on STEM teaching  PD8: Lack of exposure to STEM teaching  PD3: Inadequate quantity of STEM resources  PD2: Inadequate availability of quality instructional materials on STEM teaching  PD1: Inadequate quantity of STEM instructional materials  PD6: Limited time for teachers to perform STEM teaching  PD10: Inadequate and outdated lab facilities  PD9: Large class size  PD5: Limited time for teachers to plan and prepare for STEM teaching | 0.787  0.778  0.765  0.747  0.740  0.718  0.713  0.706  0.688  0.678 |  |  |  |
| Factor 2: *Knowledge on interdisciplinary and related pedagogical strategies (KN)*  KN5: STEM education can be implemented across all levels.  KN6: STEM education is the integration of STEM domains into science and mathematics subjects.  KN7: STEM education equips students with the knowledge to participate in technology design and creation.  KN2: STEM education prepares students for their future careers.  KN4: Problem-, project-, and inquiry-based approaches are used in STEM education.  KN8: STEM education equips students with skills to participate in technology design and creation.  KN3: STEM education requires students to draw knowledge from science and mathematics to apply in technology and engineering problems. |  | 0.840  0.788  0.786  0.774  0.747  0.719  0.708 |  |  |
| Factor 3: *Teachers’ self-efficacy beliefs to perform STEM teaching (PE)*  PE1: I am responsible for the student's achievement.  PE2: I can overcome students’ inadequacy in STEM fields through good teaching.  PE3: I am continuously finding better ways to deliver STEM education.  PE4: I am confident in my ability to deliver STEM education. |  |  | 0.814  0.797  0.736  0.732 |  |
| Factor 4: *STEM teaching practices (STP)*  STP3: I regularly observe other teachers’ STEM education and approaches.  STP4: I always share my ideas with others.  STP2: I use problem- and project-based approaches to execute STEM education.  STP1: STEM education forms an integral part of my everyday teaching.  STP8: I use lecturing and interactive instructions in STEM lessons  STP5: STEM teaching engages students to work in groups.  STP7: STEM teaching involves students solving daily problems.  STP10: Questioning is an important component of STEM teaching. |  |  |  | 0.719  0.698  0.798  0.715  0.631  0.622  0.605  0.515 |
| Cronbach’s alpha | 0.849 | 0.803 | 0.810 | 0.833 |
| Eigenvalues | 12.651 | 4.100 | 2.516 | 1.816 |
| Variance explained (%) | 38.335 | 12.424 | 7.623 | 5.504 |
