# Supplementary material for: Designing instrument to measure STEM teaching practices of Malaysian teachers
Source: PLoS One. 2022 May 20;17(5):e0268509. doi: 10.1371/journal.pone.0268509 (PMC9122257; doi:10.1371/journal.pone.0268509)
Supplement: S3 Dataset — (DOCX) [file pone.0268509.s006.docx]

**Qualitative Interview Data**

| **Interview Content** | **Coding** | **Category** |
| --- | --- | --- |
| A1: OK. STEM education is student-centered education where the students need to explore, find information, work together in groups to explore a Science or Mathematical concept. Then they need to produce a project or apply this new knowledge to create technological tools.  In my opinion, STEM Education is more comprehensive. It needs to contain all of the above mentioned domains, none of which can be excluded. | STEM helps students to solve daily life problems  Misconception that STEM is about technology  STEM activities  STEM Domains/  Definition | Knowledge on STEM Education  Knowledge on STEM Education  Knowledge on STEM Education  Knowledge on STEM Education |
| A2: Actually, it needs to be conducted simultaneously. Everything is interconnected and there needs to be continuity. It also cannot be seen as a single subject. That is, it can be applied, can be introduced during teaching and learning, meaning that during teaching and learning Science or Mathematics, STEM needs to be integrated.  Yes, throughout the lesson, teachers need to introduce STEM. | Difference between STEM and | Knowledge on STEM Education |
| A3: To ensure the students have necessary skills, hands-on, mind-ons. They can explore something unrestricted and not limited to a certain scope. They are also given the skills to learn a broader and deeper topic.  Students are more skilled in solving the problems they face.  Certainly if they are equipped with knowledge as well as methods to analyze problems then apply this knowledge, so in whatever field they are involved, they will have the skills needed to make their own decisions. | STEM to develop skills  STEM Careers  STEM to solve daily life problems | Knowledge on STEM Education  Knowledge on STEM Education  Knowledge on STEM Education |
| A4: There are various activities such as games, video filming, experiments and project based learning (PBL).  Actually it can be revised again. In Science Experiment, students are more likely to prove something but not to explore. Before they conduct the experiment, they have the answers and have been manipulated by the opinion and perspective of the teacher. They cannot analyze the problem. While in STEM Education, it is more about how the students utilize the skills and abilities to explore, understand the topic and explore something new. | STEM activities and approaches  STEM to develop skills | Knowledge on STEM Education  Knowledge on STEM Education |
| A5: Teachers need to equip themselves with 21st century teaching methodology, meaning that they need to be advanced and equip themselves with knowledge to expose STEM to students. But now, I have no direction, no exposure or given the right direction to implement it. So in fact, I faced some constraints.  For example, the education system. I am Physics teacher. In my field, I am too focused on finishing the syllabus. Then, the assessment method is more exam-oriented where the students need to reach and achieve certain objectives or level of measurement. Besides, students are more interested in getting A’s rather than acquire skills. Perhaps they think that it is not important, no scores are given, and there is no reward so they do not have a serious attitude towards its implementation. That's what I see from the point of view of a teacher in this school. | Teachers’ willingness in improving their skills  Lack of exposure  Additional workload for teachers | Efficacy in practicing STEM Education  Difficulties in practicing STEM Education  Difficulties in practicing STEM Education |
| A6: First of all, teachers need to be more open minded to accept all the possibilities. Teachers need to read more, find more information from the media for example from YouTube, Google, conduct more activities, bring the situation to class and provide many materials that can help students towards understanding the STEM approach. But the problem is it takes time. In some schools, the teachers are burdened with a lot of ad-hoc tasks for example writing reports, preparing instruments for assessment and jobs that require multiple filing that takes up much time. Based on the sharing session during a dialogue session at RECSAM, a suggestion from a Japanese education leader, during the afternoon session, students spent more time doing something to help them understand, investigate, collect information and create tools. Then they will present and promote the material to the community. In my school, the afternoon sessions are filled with extra classes to help students score in all subjects. They are not given the opportunity to apply knowledge into daily life or to appreciate and use the knowledge for the future. | Teachers’ willingness in improving their skills  Additional workload for teachers | Efficacy in practicing STEM Education  Difficulties in practicing STEM Education |
| A7: I hope trainee teachers in universities are well trained, emphasizing on creating the role model for STEM. It's not just about traditional methods to answer questions, but they should have the skills to develop STEM knowledge in the future. So, while studying in universities, they need to be given real exposure and essential skills to guide students towards STEM application.  This is very important, but due to time constraints, they could not spend too much time in this field, but it is obligatory for the teachers to spend few hours a month in improving their teaching method. |  |  |

| **Interview Content** | **Coding** | **Category** |
| --- | --- | --- |
| A1: In my opinion, STEM Education is, if we look in English, STEM is Science, Technology, Engineering and Mathematics. So, STEM must be related to the teaching of Science, Mathematics especially in schools. So, I feel STEM is something related to cultivating scientific values ​​among students.  It is actually important. So that's why I think the Ministry of Education has introduced the subject of RBT and ASK. So now we can say that STEM is covering these four areas.  Actually it is the integration of many subjects. So we cannot focus on only one subject in it. | STEM Domains and Definition | Knowledge on STEM Education |
| A2: If we look at the old syllabus, maybe the STEM element is already in the syllabus, but we did not implement it. But in the current syllabus, it emphasized on STEM exposure to the students. For example, in Science, we conduct science experiments without proper reports but now if we do an experiment, it has a report with a slightly different presentation. Another example, in Mathematics, the questions used to be straight-forward but now the questions are integrated with HOTS (High Order Thinking Skills) and also STEM concepts as well. | Difference between STEM Education and traditional Science and Mathematics subjects | Knowledge on STEM Education |
| A3: In terms of implementation is less but perhaps in the future, I will try to apply it.  Formerly, the KSSM syllabus is different than the current DSKP. Three years ago when we were still using KSSM, the students were not exposed to current performance standards. Whereas, when I look at the DSKP, the standard of performance is there with STEM integration, so it might take some time for the teachers to explore because the teachers now need to do other activities and these causes me to not have enough time to implement STEM among students.  No, no, it does not add to the burden, but, as teachers require training to understand STEM. STEM is a new element in education. Although there was STEM previously but it was not fully emphasized like now. | Time Constraint | Difficulties in practicing STEM Education |
| A4: Never, because I got to know about STEM from my friends and colleagues. At first I was confused with STEM but later I manage to get some information about STEM but that's not enough to apply in teaching and learning process as well as exposing STEM to students. So as a teacher, I think teachers need training in STEM especially in improving knowledge about STEM. | Lack of training  Lack of exposure | Difficulties in practicing STEM Education |

| **Interview Content** | **Coding** | **Category** |
| --- | --- | --- |
| A1: STEM Education is actually educating the students in four disciplines of Science, sorry four disciplines, that would be Science, Technology, Engineering and Maths, ok, it actually creates critical thinking as well as increase students literacy in science, because when you talk about literacy in science, the number of students who are actually enrolling in the Science stream is declining in the past few years. This is based on the PT3 results that we get, ok, most of the students, they don’t dare to enter, they don’t have the courage to enter Science stream they have the perception that in PT3 itself the Science subject is difficult, so how am I going to score well in the Science stream, so that’s why the number of students have been declining. So, by having STEM, is actually not only for Science stream, its actually for me it can be found in all the subjects actually, mostly Maths, Physics, Chemistry, Biology, what else, Engineering, and normal Science. All these have started in the beginning, from Form 1 itself you can find STEM, so this is not something new. | STEM Domains and Definition | Knowledge on STEM Education |
| A2: Ensuring successful STEM Education, well a teacher should be flexible first of all, must be responsive to students needs in learning, meaning students who are learners, they tend to ask lots of questions, and the teachers should be ready to answer the questions. And one more things, the questions that the teacher posed should be more towards problem solving type of questions. There shouldn’t be any right or wrong answers, there should be subjective questions where the students can give any answers but if the answer is incorrect, guide the students, why it is incorrect and try to guide the students to a correct answer. And lots of hands-on activities, it’s very important at this time. STEM Education is actually involving lots of hands-on activity so by doing that, you are actually guiding the students for a better learning environment. | Teachers as facilitators  STEM activities and approaches | Efficacy in practicing STEM Education  Knowledge on STEM Education |
| A3: School lab infrastructure, well, when you look at the schools, the new schools, let say 15 to 20 years ago, let say 20 years old school, the lab is quite big, it’s very spacious and the design also nice compared to schools older than 20 years old. So, the new labs are well designed where the students can carry out the experiment in a proper and better manner, they can work as a team, as a group, in a conducive environment. If you want to learn something, the environment must be permitting; it should be in a conducive manner for the students to learn. But then, schools that are old schools, you don’t get all this. The lab is small, only can accommodate certain number of students. One more thing when you talk about STEM, the allocation that you get each year is also important. The allocation from the government, because in order for STEM to be successful, there should be enough teaching aids, because STEM, lots of teaching aids are used for STEM. So, if you don’t have proper teaching aids, how are you going to teach using STEM? So the allocation is very important. | Lack of infrastructures  Lack of resources | Difficulties in practicing STEM Education  Difficulties in practicing STEM Education |
| A4: Am I confident? No, you know why? Because, I’m a Bio teacher, if you ask me about Bio, I know, I’m very confident with my content level in Bio and my teaching ability. But when it comes to STEM, ok like I say, STEM, Science, Technology, Engineering and Maths, I am not good in those divisions, ok, those are not my field. Suddenly if you give me a question where you include all the STEM elements, I may have some problems when it comes to Technology, Engineering and Maths, ok, so basically I am not saying I can’t teach, I can teach if you give me proper training, after training you provide me with a module to follow I can definitely teach STEM. I need that confidence. Not only for me, for so many other teachers as well, give them proper training, longer hours of training, boost their confidence level so that when they go back to school and teach their students, they will be more confident as well. | Teachers; confidence  Lack of training | Efficacy in practicing STEM Education  Difficulties in practicing STEM Education |
| A5: How often? I don’t.  Ok, because I’m teaching Form 5 students, they will be sitting for public exam in November, right, so I also follow the curriculum strictly. So in the curriculum, there is no mention about STEM. I’m talking about the current curriculum, there is nothing mentioned about STEM there, so I don’t waste my time, because my main focus is to prepare the students for the public exam, because there is not questions regarding STEM in public exam questions as well. That’s the thing. So my focus is more towards preparing the students for the exam only, I don’t focus on STEM activity. | Time constraint  Additional workload for teachers | Difficulties in practicing STEM Education  Difficulties in practicing STEM Education |

| **Interview Content** | **Coding** | **Category** |
| --- | --- | --- |
| A1: In general, when I hear the word STEM, it refers to how I integrate STEM learning which refers to the words Science, Technology, Engineering and Mathematics. So it is how I implement these STEM elements in my teaching, it does not matter whether I teach Science, Chemistry or any other subject, I need to integrate STEM into the subject.  Yes. STEM does not necessarily mean that all the elements must be present. In America, for example, the job availability for the people there, because they experience it, the element that is stressed is STEM, because the percentage of job opportunities is higher in STEM related fields. They have been integrating STEM for a long time in their education system. So, not necessarily in one subject there should be Science, maybe we can just apply technology, maybe we just apply Mathematics, there is a wide scope covered in STEM, not restricted to one subject only. It depends on the suitability of the subject. There may be a subject that we cannot fit in engineering. | STEM Domains and Definition  STEM Careers | Knowledge on STEM Education  Knowledge on STEM Education |
| A2: Yes, when you talk about this activity, it is more focused on students, such as project based learning. We conduct a project, for example like in Form 1,we have to create a water filter. Therefore consequently, students are exposed to engineering aspects, Science was already there, and technology, what else, and Mathematics too. Maybe in what way and how much to use. So it covers overall. We do not need to have a learning framework since it has been implemented indirectly. | STEM activities and approaches  Engineering as important aspect in STEM | Knowledge on STEM Education  Knowledge on STEM Education |
| A3: For me no problem because I teach Science subjects, there may be problems for certain teachers who are not in this field. So for a science teacher like me, I do not face any problem. |  |  |
| A4: In terms of acceptance, students really like it because it is more student-oriented, where the student creates and explores. At first, they will explore, they will be told to be creative, so that's where we emphasized on engineering. When the students are exploring, they enjoy it. We do not restrict to one product only. Students are free to create anything, so at that time, their learning will be more meaningful. When it is meaningful, they will appreciate and will remember it when leaving the class.  When we conduct project based learning, we gave them group discussions. But when we conduct presentations, we will evaluate based on individuals as well. In one group, not all the members are capable of presenting despite having ideas. At one point, we will have to look for the cooperation in the group and at a certain time we have to see how the individual itself acts in the group. |  |  |
| A5: In my view, I do not think I face any problems to implement STEM, it can be done because it has been applied since I started teaching. So this thing is not a new thing, it has been implemented for a long time, so no problem for me.  Yes, I am sure I can do it. There is no problem to implement STEM but we still need courses. We need a course to make it more stable and to improve how STEM should be implemented. So far no problem, I can do STEM in any subject. | Teachers’ confidence  Lack of training | Efficacy in practicing STEM Education  Difficulties in practicing STEM Education |

| **Interview Content** | **Coding** | **Category** |
| --- | --- | --- |
| A1: Integration of Science, Technology, Engineering, Mathematics in our teaching.  Ok, it’s about how we teach the activity, in my field, in Science, how we are teaching Science to help them in their life time, how are they going to integrate Science that they learn in school into their lifestyles, how they use it in their daily life.  Because I’m teaching Science, that’s why I am focusing more on Science only, because for technology in Science, I think, for my students, most of them are Art Stream students, so they will learn only Science, they didn’t take Bio, Chemistry and Physics. | STEM Domains and Definition  STEM to solve daily life problems | Knowledge on STEM Education  Knowledge on STEM Education |
| A2: Actually not for every class because, for the first and second class, they are very good students, they can do all the activities, for example, Project Based Learning activities. They will do the activities on the time. After the lesson, when we get the feedback, they will give the feedback. But for the weak students, they are unable to do the activities regarding to STEM and they don’t understand on how to do the activities.  Yes, yes.  For the first class, they manage to get to do the activities that we give. They can do the group work, they can solve the problems, most of the activities we give are on how to solve problems.  I think some of them will.  A3: Because, there are some other factors like the materials. The materials that we need for STEM, some of the materials are expensive and some are hard to find for these activities. Because some of the activities we conduct at a precise timing, we cannot obtain the materials needed at that particular time.  Yes, yes. | STEM Careers  Lack of resources | Knowledge on STEM Education  Difficulties in practicing STEM Education |
| A4: Of course not. In our school, we are lack of the infrastructure, such as, our school is not even provided with projectors. We buy them on our own. Some of the teachers buy their own projectors. So, these are the factors that we have to face, the problems in conducting STEM activities in our school. | Lack of infrastructure | Difficulties in practicing STEM Education |
| For STEM Education, we need the government to support with some infrastructure to conduct these activities. Without these infrastructures, we cannot conduct all the activities | Lack of infrastructure | Difficulties in practicing STEM Education |

| **Interview Content** | **Coding** | **Category** |
| --- | --- | --- |
| A1: STEM..S-T-E-M right? STEM as what I know, it is a type of a combination, a kind of subject that combines the elements of Science, Technology, Engineering and Mathematics. It is a concept, I am not sure of the word but I was told to use STEM when teaching Mathematics.  No, it is not a single subject or a syllabus and there is not a single textbook titled STEM.  Domain? Which one? Between Science and Technology? I think cannot. I do not think so. It is not complete if we lose one of the domains. Then we cannot refer to it as STEM right? | STEM Domains and Definition | Knowledge on STEM Education |
| A2: Hmm..I try my best to do all this because it is important now. I was told to use STEM during teaching and learning, but I am also urged to finish the syllabus two weeks before the examination or two weeks before the test. So if we really want to do it during teaching and learning process, it takes time. Students will be working in groups, the class will be noisy, and the teacher needs to control the class. For example, in my class, there are more than 40 students. So, it is impossible to apply STEM every day. Not all topics can use STEM.  Hmm , yes. In some topics I will use STEM.  No, cannot. There are certain topics that can’t be integrated with STEM. | Time constraint  Large class size | Difficulties in practicing STEM Education  Difficulties in practicing STEM Education |
| A3: It depends on the class. It depends on students’ level of proficiency and differs in every class. Some of them are really interested; interested to know more, eager to know the answer, interested to know the reason and cause, to know the connection technology has in their life. This is when students are really interested in Science and Technology. For those who are not interested, no matter what activities we give them, they will not do. But at one point, for the students who are interested in the activities, we will have to focus more on that group and it really takes time. We've tried it and one teaching and learning session is not enough.  Honestly, I faced many problems in terms of preparation. In order to prepare teaching materials, we need to have a lot of teaching aids. Working as a teacher now, we do not just teach 100% but we need to do other ad-hoc works as well, right.  I would need to attend a course for that. With the guidance from experts from that field, it is possible.  **Probe:** What about reference materials? Do you have problems finding reference material?  The reference material, in my place, it is limited. Sometimes I need help from friends in the Peninsular. | Lack of resources  Additional workload for teachers  Lack of resources | Difficulties in practicing STEM Education  Difficulties in practicing STEM Education  Difficulties in practicing STEM Education |
| A5: I am not 100% confident, but at least I am half confident. It is because I have knowledge in Science field, but I cannot say I am 100% confident because I have not attended any courses, have not been informed of the proper materials to be used and I am not aware of the difficulty level that is appropriate with my students’ proficiency. | Teachers’ confidence | Efficacy in practicing STEM Education |

**Respondent 8**

| **Interview Content** | **Coding** | **Category** |
| --- | --- | --- |
| A1: STEM Education in my opinion actually is about the lesson which is a combination of Science, Technology, Engineering and Mathematics.  A lesson which is conducted with a combination of these four elements. | STEM Domains and Definition | Knowledge on STEM Education |
| A2: Most of the time. Actually, for example, normally I will ask the students in my daily lesson, I will ask the students to use their mobiles to search for information and gather the information into a power point presentation, using a laptop. The laptops are provided by school. So they will present their lesson using power point. But the points and information, they gather using their mobiles via the Internet. By this, easy for them to get extra information, combining with the information given by myself, but they get the extra information from the Internet.  Most of the time.  Ok, one of the latest activities that I used in Mathematics, I asked my students to construct a 3D dimension of polygons, for example cuboids, cubes, pyramids and cones. What I did was describing to them the 2D dimension and taught them the formula for the geometry polygons. Example for cubes, I was teaching them the formula used to calculate the volume of cubes. So, by just demonstrating them the 2D dimension, I use the power point and internet to show them how to construct the shape of the 3D dimension. From that they need to find what is the length, width and then the height of the polygon and the need to understand the formula. So, that’s one of the lessons that I use technology in Mathematics. Other than that, in Biology, they need to participate in groups. Mostly I use technology in Science and Mathematics.  All the activities I asked them to conduct in groups so that it would be easy for them to communicate and share information.  Actually, the group is formed by themselves. Normally I just tell them how many groups, how many members in the group, so, they will form their own group.  Ok, like the mobiles, normally I will prepare the mobile because we have extra mobiles from Ministry Department, so each group will be given a mobile. They are only small groups, about five or six members in a group, so they use the mobiles to search for information and the WiFi is also provided to them by the teacher, so, there will be five groups, so only five devices given and the laptops will be given by the school, because we have labs with computers. So they will use the computers to do their work and present their work via Power Point.  won’t say enough, still lack of resources. Lack of devices. For example, in my class, in one group there are almost 3-5 students who will be sharing one device and one mobile. As for my concern, that insufficient, so I won’t say enough. | Misconception that STEM is about technology  Lack of resources  Time Constraint | Knowledge on STEM Education  Difficulties in practicing STEM Education  Difficulties in practicing STEM Education |
| A4: Based on my six years of experience in Sabah, I was posted here in 2013. So far, there are no extra courses given regarding STEM.  Because I was doing my masters, one of my papers was regarding technology usage. So, I was taught by some lecturers about STEM and how the activities are conducted. For example, I learnt how to use Kahoot! by learning on my own and some are taught by teachers here. Most of the time it is from my own learning. | Lack of training  Lack of exposure  Lack of exposure | Difficulties in practicing STEM Education  Difficulties in practicing STEM Education  Difficulties in practicing STEM Education |
| A5: For me, I just want the students to learn daily basics. I just want them to learn this STEM. I want them to find it easy to understand the lesson and make it useful in their future careers. By using skills they have learnt especially in Maths and Science and connect them to real world problems.  Yes, to have better careers in future. | STEM to solve daily life problems  Teachers’ as facilitators  STEM Careers  STEM to develop skills | Knowledge on STEM Education  Efficacy in practicing STEM Education |
| A6: So far, I can give you the percentage, maybe 60% I am confident to teach STEM in my lesson. Because I still think we have lack of resources, I mean materials, tools and devices. So maybe if I have enough tools and maybe extra training by the Education Department, I will be 100% confident to use STEM in my lesson. | Teachers’ confidence | Efficacy in practicing STEM Education |
